# First robotic telesurgery applied to bariatric surgery in Latin America: technical fundamentals, operational challenges and perspectives in clinical practice

**DOI:** 10.1590/0102-672020260000010e1939

**Published:** 2026-06-22

**Authors:** Paulo Afonso Nunes NASSIF, Osvaldo MALAFAIA, Gualter Lisboa RAMALHO, Augusto de ALMEIDA, Rafael Mourato Inácio da SILVA, Mariano PALERMO, Leandro Totti CAVAZZOLA, Marcelo de Paula LOUREIRO

**Affiliations:** 1 Scolla Ensino – Campo Largo (PR), Brazil.; 2 Faculdade Evangélica Mackenzie do Paraná – Curitiba (PR), Brazil.; 3 Hospital Alberto Urquiza Wanderley – João Pessoa (PB), Brazil.; 4 Hospital National Profesor Alejandro Posadas – Buenos Aires, Argentina.; 5 Universidade Federal do Rio Grande do Sul, Hospital das Clínicas, General Surgery Service – Porto Alegre (RS), Brazil.; 6 Universidade Positivo – Curitiba (PR), Brazil.

**Keywords:** Obesity, Telesurgery, Robotic surgical procedures, Bariatric surgery, Organizational innovation, Obesidade, Telecirurgia, Procedimentos cirúrgicos robóticos, Cirurgia bariátrica, Inovação organizacional

## Abstract

**Background:**

The use of robotic systems in bariatric surgery has reached a stage of maturity that supports its application with consistent results in the management of severe obesity. At the same time, the improvement of communication networks and digital support bases has renewed the debate on telesurgery as an alternative to extend the reach of specialized surgical care, particularly in contexts marked by the unequal concentration of highly complex services.

**Aims:**

To present the first teleassisted bariatric robotic operation in Latin America between two states of Brazil separated by 3,200 km, in order to demonstrate its operational capacity in real time.

**Methods:**

Vertical gastrectomy was performed with two separate teams: one performing the procedure in the metropolitan area of Curitiba (PR), and the other in the city of João Pessoa (PB), both in Brazil.

**Results:**

The procedure was performed without significant operative differences when compared to the face-to-face system and in the same way that it could have been done without the use of telesurgery.

**Conclusions:**

Within this transition process, robotic assisted bariatric surgery stands out as a strategic environment for experimentation, systematic evaluation and consolidation, giving access to new approaches, such as telesurgery, contributing to the construction of a more integrated and innovative surgical model.

## ARTICLE HIGHLIGHTS

This study reports the first teleassisted robotic bariatric surgery in Latin America, performed between two Brazilian states separated by approximately 3,200 km.

A robotic sleeve gastrectomy was completed with two teams working remotely in real time, without significant operative differences compared with conventional in-person robotic surgery.

## Summary

**CENTRAL MESSAGE:**

The gradual incorporation of sophisticated technological solutions into the surgical routine has been profoundly redefining contemporary models of health care, professional training processes, and the development of applied research. In this scenario, robotic surgery plays a central role as it represents more than an improvement on traditional laparoscopic techniques. It is about associating it with complex and integrated systems, in which high-precision mechanisms are associated with digital control platforms, instant transmission of information and advanced visualization resources, significantly expanding the technical and scientific possibilities of modern surgery with a focus on telesurgery.

**PERSPECTIVES:**

Bariatric surgery performed with robotic support goes beyond the concept of a minimally invasive technique, and becomes part of a broader movement of digital transformation of surgical practice. The articulation of these systems with telesurgery resources opens relevant perspectives for the expansion of specialized care, the development of innovative teaching strategies, and the strengthening of scientific research in the area. However, for this potential to be effectively achieved, the incorporation of these technologies must occur gradually, with welldefined criteria and supported by strict principles of safety and quality of care.

## VISUAL ABSTRACT

**Figure:**
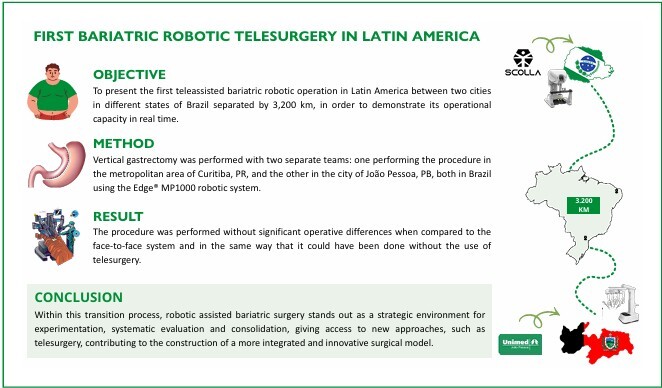

## RESUMO

**Racional:**

A utilização de sistemas robóticos na cirurgia bariátrica alcançou estágio de maturidade que sustenta sua aplicação com resultados consistentes no manejo da obesidade severa. Ao mesmo tempo, o aprimoramento das redes de comunicação e das bases digitais de suporte têm renovado o debate sobre telecirurgia como alternativa para estender o alcance da assistência cirúrgica especializada, particularmente em contextos marcados pela concentração desigual de serviços de alta complexidade.

**Objetivos:**

Apresentar a primeira operação robótica bariátrica teleassistida na América Latina entre dois estados do Brasil separados por 3.200 km, com o intuito de demonstrar sua capacidade operativa em tempo real.

**Métodos:**

Foi realizada gastrectomia vertical com duas equipes separadas: uma efetuando o procedimento na área metropolitana de Curitiba, PR, e outra na cidade de João Pessoa, PB, ambas no Brasil.

**Resultados:**

O procedimento foi efetuado sem diferenças operatórias significativas quando comparado ao sistema presencial e do mesmo modo que poderia ter sido feito sem o uso da telecirurgia.

**Conclusões:**

Dentro desse processo de transição, a cirurgia bariátrica assistida robótica destaca-se como ambiente estratégico para experimentação, avaliação sistemática e consolidação dando acesso a novas abordagens, como a telecirurgia, contribuindo para a construção de modelo cirúrgico mais integrado e inovador.

## INTRODUCTION

 The progressive adoption of highly complex technologies has structurally transformed modern surgical practice, impacting patient care. In this context, robotic surgery has consolidated itself as a paradigm shift, overcoming the idea of simple evolution of laparoscopy. It is an integrated platform that combines advanced mechanical precision, sophisticated digital command and control systems, real-time data processing and transmission capabilities, and high-definition imaging capabilities, significantly expanding the technical, educational and scientific possibilities of contemporary surgery^5,6,10^. 

 In the field of bariatric and metabolic surgery, the use of robotic systems has been gradually validated as evidence of consistent results, with high safety standards and reliable execution. These procedures show a special advantage in situations of greater technical complexity, such as reoperations, management of patients with extreme obesity, and the performance of anastomoses or sutures that require a high degree of precision. However, the impact of these technologies is not limited to improving the surgeon’s posture or refining movements. Robotic platforms represent a broader conceptual advance, introducing new possibilities in the way contemporary metabolic surgery is planned, executed, and evolved. 

 Although telesurgery was proposed at the end of the last century, its routine incorporation into clinical practice remained, for many years, restricted to specific experiences. Technical limitations related to the delay in data transmission, instability of connections, vulnerabilities in digital security, and the high cost of available platforms prevented its largescale consolidation. However, the recent evolution of communication infrastructures, with the expansion of high-speed and low-latency networks, the improvement of encryption mechanisms, and the increase in the reliability of robotic systems has significantly changed this scenario. In this new technological context, robotic bariatric surgery emerges as an especially favorable environment to reevaluate telesurgery, now from a contemporary perspective, feasible and aligned with the current demands of surgical practice^1,2,5,6,14^. 

 Considering the high worldwide prevalence of obesity, the unequal concentration of professionals with advanced training in bariatric surgery, and the systematized nature of most of these interventions, bariatric surgery performed with robotic support stands out as a particularly favorable scenario for the incorporation of telesurgery. This combination creates concrete opportunities not only to expand access to care, but also to strengthen professional training strategies and boost scientific research, consistently integrating the clinical, educational, and investigative dimensions of contemporary surgical practice. 

 The objective of this work was to present the first teleassisted bariatric robotic operation in Latin America (Source: PubMed, Scopus and Google Scholar – March/2026) between two cities in states of Brazil separated by 3,200 km, in order to demonstrate its operational capacity in real time. 

## METHODS

**Figure 1:**
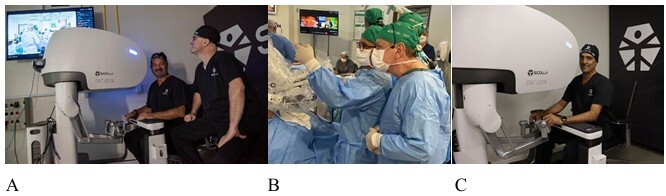
Operative times of bariatric telesurgery: (A) in Curitiba (PR), P.A.N.N (left) and L.T.C. (right) are seen in action on the console; (B) in the operating room of the Alberto Urquiza Wanderley Hospital in João Pessoa (PB) with M.P.L. (left) and A.A.J. (right); (C) M.P. handling the console.

 This procedure was performed in Brazil, with the console and surgical team in the metropolitan area of Curitiba (PR), and the patient in João Pessoa (PB). The participating institutions from Paraná and Paraíba were SCOLLA — a Surgical Training Center in Campo Largo (PR); Alberto Urquiza Wanderley Hospital/Unimed Hospital in João Pessoa (PB); and Mackenzie Evangelical College of Paraná in Curitiba (PR) (Figure 1). 

 The connectivity architecture for long-distance robotic telesurgery (3,200 km) was structured on a hybrid redundancy system, integrating internet links from local providers with a dedicated high-performance line. 

 Data traffic management was carried out by two specialized modems from the manufacturer EDGE Medical/MP 1000 (China), configured to operate in a low-latency ecosystem, where the flow is ensured by active redundancy protocols and security perimeters via firewall robust. This network configuration allowed the maintenance of propagation delay (RTT) consistently below 35 ms, ensuring the fidelity of the motor commands and the stability of the feedback real-time sensory. The convergence between low-cost infrastructure and dedicated connectivity ensured not only the economic viability of the system, but also the critical resilience required for the practice of remote minimally invasive surgery in transcontinental settings. 

### MP 1000 robotic system platform

 This was the equipment used. It is a state-of-the-art robotic surgical system, designed to perform minimally invasive procedures with precision, safety and efficiency. This equipment combines advanced technology, ergonomics, and innovative features such as telesurgery to transform modern medical practice. 

### Surgical procedure

 The procedure was submitted to the double appreciation of the Ethics Committees for Research on Human Beings of the two institutions involved, and authorization was also granted by the Regional Council of Medicine of Paraíba (PB) for the surgeon from Paraná (PR) (Paulo Afonso Nunes Nassif) to be admitted as a temporary member for a period of 90 days (protocol n^o^ 088521), in order to be able to perform the procedure remotely. The operation was performed on a 50-year-old woman, weighing 95 kg, 1.55 m tall, with a body mass index (BMI) of 39.5 kg/m^2^, who had been obese for more than 25 years, developing insulin resistance and severe arthropathy of the spine in the pre-diabetes period. Due to the pain, she had difficulty performing physical exercises and, for this reason, she underwent two neurolysis procedures in the spine. Given work difficulties and clinical intractability, she was indicated for a bariatric procedure. 

 The operation was performed in compliance with all the technical-surgical details recommended by the procedure, with no methodological changes. The operational details will be better explained in a later specific publication that will offer the technical specificities, both in what was carried out in the Metropolitan Region of Curitiba — Campo Largo (PR), and in João Pessoa (PB). 

### Sleeve gastrectomy

**Figure 2:**
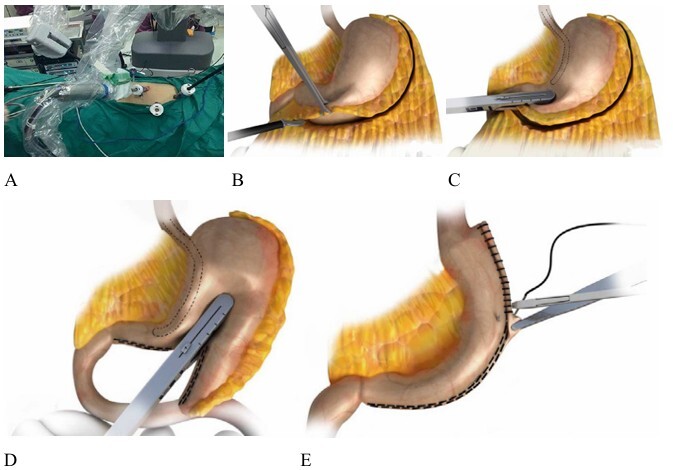
Surgical procedure: (A) demonstration of the position of the trocars; (B) devascularization of the great curvature extending from the pylorus to the esophagogastric angle with section of the coalescences of the posterior wall of the stomach; (C) antral stapling with black or green loads; (D) stapling and sectioning of the gastric body towards the esophagogastric junction; (E) final aspect of the staple line showing the tubular shape of the stomach, from the pylorus to the cardia.

 The surgical procedure followed the same robotic operative times, without telesurgery, and is summarized in Figure 2. 

## RESULTS

 The application of telesurgery demonstrated high technical-operational feasibility, allowing the full performance of the surgical procedure under adequate and reproducible conditions, without the occurrence of relevant technical difficulties or the need to adapt the previously established operative technique. All stages of the surgical procedure were conducted in a fluid manner, with adequate execution of the operative times and preservation of the fundamental principles of the surgical technique. 

 The interaction between the teams, geographically located in different states, occurred in a continuous, stable and synchronized manner throughout the procedure, evidencing consistent performance of the technological infrastructure employed. Data transmission, including operative commands and audiovisual communication, remained efficient throughout the surgical procedure, with no interruptions or noticeable latency that could interfere with the technical conduction. 

 From the intraoperative point of view, no complications, adverse events, or instabilities that would compromise patient safety or the quality of the procedure were observed. The surgical execution remained precise and controlled in all its stages, reflecting adequate integration between the local and the remote team. 

 In general, these findings show that telesurgery, under the conditions in which it was performed, presented satisfactory technical performance, with maintenance of safety, precision and quality of the surgical act, even in the face of the geographical separation between the centers involved. 

## DISCUSSION

 Recent evidence indicates that robot-assisted bariatric surgery has reached an advanced stage of technical consolidation, enabling its safe application even in highly complex situations, such as in patients with extreme obesity and in reoperations. The high degree of uniformity in the results and the ability to reproduce the operative steps play a central role when discussing telesurgery, as technical predictability is an indispensable element for the execution of remote procedures with adequate levels of safety. From this perspective, robotic bariatric surgery has unique advantages in relation to other specialties by bringing together a large volume of clinical demand, relevance in terms of public health, and well-structured surgical protocols, configuring itself as an especially favorable field for the development and application of telesurgical strategies. 

 From the perspective of digital infrastructure, the accelerated evolution of communication systems has decisively changed the telesurgery scenario. The expansion of fiber optic networks, associated with the development of fifth and sixth generation mobile technologies, has overcome historical limitations related to the speed and stability of data transmission. Recent evidence indicates that very low levels of latency and high security in communication can already be achieved consistently, including in developing country contexts, as demonstrated by experimental initiatives conducted in Brazil. This panorama supports the understanding that the contemporary challenges of telesurgery go beyond the purely technological field, starting to include factors of an organizational, normative, and ethical nature, which become central to its responsible and sustainable implementation. 

 The rapid evolution of communication technologies has promoted a substantial change in the potential application of telesurgery. The advancement of fiber-optic infrastructures, combined with the consolidation of fifth-generation mobile networks and the development of sixth-generation mobile networks, has significantly expanded the capacity for real-time data transmission^3,4,6,7^. Recent investigations have shown that high standards of stability and minimal delays in communication can already be achieved, even in environments with less technological maturity, as illustrated by experiences conducted in Brazil^1^. This set of evidence supports the understanding that the current obstacles to the expansion of telesurgery go beyond the technical domain, encompassing institutional, normative, and ethical challenges, which play a central role in the contemporary debate. 

 In the field of medical education, the convergence between robotic platforms and telesurgery resources introduces structural transformation in the processes of training in surgery. Traditional approaches, centered on the continuous face-toface supervision of specialists, are now expanded by remote monitoring mechanisms, such as teleproctoring and telementoring, favoring the dissemination of knowledge and the attenuation of regional disparities in professional training^6,13,14^. In addition, robotic systems offer the possibility of recording and analyzing objective parameters of the surgical procedure, allowing the measurement of technical performance, the analysis of learning curves and the development of educational research based on quantitative indicators, overcoming the limitations inherent to the predominantly subjective evaluation of methods used in the past^5^. 

 Ethical and normative aspects play a decisive role in the debate on the adoption of telesurgery. Evidence from systematic analyses and international positions converge in indicating that its safe incorporation depends on the existence of welldefined regulations, with precise delimitation of professional responsibilities, safeguards for the confidentiality of information, and formal criteria for qualification and accreditation. The lack of these institutional references tends to weaken the social acceptance and legitimacy of telesurgery, even in the face of proven technological capacity. In this scenario, progressive implementation strategies, initially based on hybrid models with structured supervision, are more compatible with the principles of patient safety, professional responsibility, and adequate clinical governance. 

 From the point of view of justice in access to health care, telesurgery emerges as a tool with significant social impact, by creating the possibility of offering specialized procedures in distant locations or in need of qualified professionals. However, for this benefit to materialize in an effective and sustainable manner, it is essential that its implementation be accompanied by coordinated public policy actions, adequate allocation of resources in infrastructure, and training programs that prevent the deepening of technological disparities between reference centers and less assisted areas. 

 In summary, the integration between robotic systems, advanced connectivity infrastructures, data analysis tools, and artificial intelligence applications points to a scenario in which telesurgery is not configured as an isolated objective, but as a component of a broader movement to digitize surgical practice. This process tends to produce structural and lasting impacts on patient care models, professional training, and scientific production. 

### Perspectives

 The development horizon of bariatric surgery performed with robotic support, especially when associated with telesurgery, points to a favorable scenario^2,4,6^. The expansion and diversification of the robotic systems market, stimulated by greater competition among manufacturers and continuous technological innovation, tend to generate progressive cost reductions and facilitate the diffusion of these platforms^2,9,11^. In addition, the evolution of communication infrastructures, characterized by more stable transmissions and increasingly shorter delays, contributes to making telesurgical applications closer to daily clinical practice, reinforcing their viability in the medium and long term^4,6,8^. 

 In the context of professional training, there is a growing trend towards the incorporation of integrated educational strategies, which combine simulation environments, robotic platforms and distance monitoring by specialists^5,6,14^. These hybrid models tend to occupy an increasingly relevant space in medical residency and graduate programs. In this context, telesurgery emerges as a strategic instrument to expand access to advanced training, contributing to reducing regional asymmetries in the qualification of professionals, particularly in countries marked by large geographic and structural disparities^6,14^. 

 More recent experiences have demonstrated, in a concrete way, the applicability of this educational paradigm. The emergence of robotic surgery training solutions that combine teleoperation, high-fidelity simulators, and mirrored digital models of the surgical environment has enabled the expansion of specialized training, while reducing institutional burdens and offering standardized scenarios for scientific investigation and validation of new technologies. This set of tools signals consistent progress in the structuring of hybrid training environments, integrating teaching, research, and innovation in the field of robotic surgery and telesurgery^13^. 

 In the scientific field, the articulation between robotic surgery systems, advanced analytical tools, and artificial intelligence resources has significantly expanded the scope of surgical investigations^5,6^. This integration enables more precise approaches to the evaluation of technical performance, the consolidation of operative patterns, and the objective analysis of clinical results. In this context, bariatric surgery plays a central role in the advancement of translational innovation, since it combines high frequency in care practice with broad metabolic impacts, becoming a privileged scenario for the development and validation of new technological strategies^2,9,11^. 

 Thus, bariatric surgery performed with robotic support should not be interpreted only as an additional technical resource. It is part of a dynamic and interconnected digital environment, in which different technologies converge to redefine the ways in which surgical procedures are performed, the processes of professional training, and the strategies for the production of scientific knowledge^4,6,12^. 

## CONCLUSIONS

 Even though technological, financial, and regulatory obstacles still restrict the full dissemination of telesurgery, the current panorama reveals a gradual movement towards more articulated models of clinical care, professional training, and scientific production. Within this transition process, roboticassisted bariatric surgery stands out as a strategic environment for experimentation, systematic evaluation, and consolidation of these new approaches, contributing to the construction of a more integrated and innovative surgical model. For the academic medical community, understanding this movement is essential not only to keep up with technological evolution, but also to actively participate in the construction of more accessible, safer, and more accessible surgical models. 

## Data Availability

The datasets generated and/or analyzed during the current study are available from the corresponding author upon reasonable request.
